# Comments from the incoming Editor

**DOI:** 10.1098/rstb.2023.0002

**Published:** 2023-04-10

**Authors:** Richard Dixon

**Affiliations:** University of North Texas, BioDiscovery Institute and Department of Biological Sciences, Denton, TX 76203

I am excited to assume the role of Editor of *Philosophical Transactions B* following the end of the Editorship term of Professor John Pickett CBE, FRS, with the last issue of 2022. I have known John for many years, and his infectious engagement with all things scientific very much personifies the interdisciplinary focus of the journal. He will be a hard act to follow, although I suspect we will be in touch a lot!

After taking partial retirement from the University of North Texas in October 2021, I felt that I had the time to be involved in more editorial work. But for what journal? My three years as Editorial Board member for *Phil Trans B* made me realize that working with this journal would be a very different experience from handling manuscripts for most specialist journals, or even a general interest journal such as *PNAS*, on whose board I currently serve. There is much to be said for learning new things as you get older, and the broad, interdisciplinary scope of *Phil Trans B* will certainly make sure that happens.

For those who do not know me, just a little about my background. I am a plant biochemist who graduated from Oxford, did two years postdoctoral work in Cambridge, and then spent 9 years on the faculty in Biochemistry at Royal Holloway College, University of London. In 1988 I threw caution to the wind and assumed the position of Director of Plant Biology at the Noble Foundation, a private research institute in Ardmore, Oklahoma, dedicated to promoting good agricultural practices. This turned out to be a very good decision, and for 25 years the Foundation was a wonderful place for both basic and applied plant research. It was there that I saw first-hand the power of interdisciplinary research to translate basic science to benefits for the ultimate end users, the farmers and ranchers who were supported by the Foundation's Agricultural Division. In 2013 I left the Foundation to become Distinguished Research Professor in the Department of Biological Sciences at the University of North Texas, joining a group of excellent plant scientists and founding the BioDiscovery Institute (https://bdi.unt.edu/) in 2015. I am now Professor Emeritus at UNT, maintaining a much smaller laboratory mainly remotely from my home in Tulsa, Oklahoma.

My research interests centre on the biochemistry, molecular biology and metabolic engineering of plant natural product pathways and their implications for agriculture and human health, and the engineering of lignocellulosic biomass for the improvement of forage and bioenergy feedstocks. Over the years, I have worked in several large interdisciplinary research teams addressing questions from overcoming biomass recalcitrance for processing to fuels and chemicals to understanding metabolic conversions of dietary plant flavonoids in relation to amelioration of cognitive dysfunction. I have enjoyed and appreciated how collaborations beyond one's own area of expertise or even interest can result in gains in knowledge and understanding that would not otherwise occur, and such synergy is clearly a major feature of *Phil. Trans. B*.

My first and only experience in publishing in *Phil. Trans. B.* was an article I co-authored in 1986 in the proceedings of a discussion meeting on differential gene expression in plants. Just being there at the Royal Society with many of the leaders of the field was an amazing experience for a new faculty member. Re-reading the article now makes apparent the incredible advances in technology in the biological sciences over the past 30–40 years. Back in 1986 we were asking the right questions but had limited tools to help us answer them. Now we seem to have a different problem—the age of ‘big data' has made it possible to generate, both rapidly and cheaply, far more information than one could have dreamed of back in the day. In some cases this has led to spectacular advances, but it can also lead some into blind alleys in which the wood is not seen for the trees. I very much like the focus of *Phil. Trans. B* on questions of deep biological significance.

My philosophy for the journal is simple—to maintain the high standards of quality, thematic diversity, author/editor diversity, and international collaboration that the journal currently enjoys. *Phil. Trans. B* is clearly seen as the premier place for review articles and themed issues by a number of communities and has an enthusiastic user/reader base in these areas that should be maintained. I do not have a specific agenda for changes in direction, other to than to ensure that we actively encourage issues across the whole spectrum of interest areas covered by the journal, including the climate : agriculture interface mentioned by Professor Pickett in his concluding editorial.

The present uncertain political climate further highlights the value of a journal such as *Phil. Trans. B*. International collaborations are suffering in some cases from heightened suspicion of scientists' intentions from their representative governments. More isolationist policies in many countries have reduced funding for international collaborations. These issues highlight the need for the journal to maintain, or increase, the diversity of nationalities in its thematic issues; even if transfer of materials and personnel across international boundaries is undergoing a reduction, transfer of ideas and synthesis of new concepts among the broader scientific community should be free and receive continued encouragement. Furthermore, as the climate crisis and changing political and social norms affect, in different ways and extents, countries and societies across the world, the journal should continue to encourage scientists from under-represented countries to participate in the themed issues.

I am looking forward to working with the excellent Editorial Team at the Royal Society and also to taking the opportunity of getting back to visit my home country after the restrictions of the Covid pandemic. I urge readers to come forward with suggestions for theme issues across the whole subject area of the journal—we are always looking for new topics and all scientists are welcome to submit a proposal. Please visit the journal's website for more information.

